# Intrapericardial Bronchogenic Cyst: An Unusual Clinical Entity

**DOI:** 10.1155/2014/651683

**Published:** 2014-12-15

**Authors:** Murat Ugurlucan, Omer Ali Sayin, Matthias Felten, Didem Melis Oztas, Mehmet Semih Cakir, Mehmet Barburoglu, Murat Basaran, Ufuk Alpagut, Enver Dayioglu

**Affiliations:** ^1^Department of Cardiovascular Surgery, Istanbul Faculty of Medicine, Istanbul University, Millet Caddesi, Capa, Fatih, 34390 Istanbul, Turkey; ^2^Department of Radiology, Istanbul Faculty of Medicine, Istanbul University, Millet Caddesi, Capa, Fatih, 34390 Istanbul, Turkey

## Abstract

Mediastinal cysts are extremely rare clinical disorders. They usually have a pericardial origin. In this report, we present a 27-year-old male patient with a mediastinal bronchogenic cyst together with clinical presentation and management of the pathology.

## 1. Introduction

Cystic lesions may occur in the mediastinum; however, they are rare. They can have pericardial, enteric origin or bronchogenic origin in most of the cases [1, 2]. Usually the patients are asymptomatic, and when the lesion leads to symptoms, since it was first reported in 1959 by Meyer [3], the standard therapy is the resection of cyst. The literature seldom reveals reports of intrapericardial bronchogenic cysts.

Here, we report clinical presentation and management of a mediastinal bronchogenic cyst in a 27-year-old patient.

## 2. Case Report

**Figure 1:**
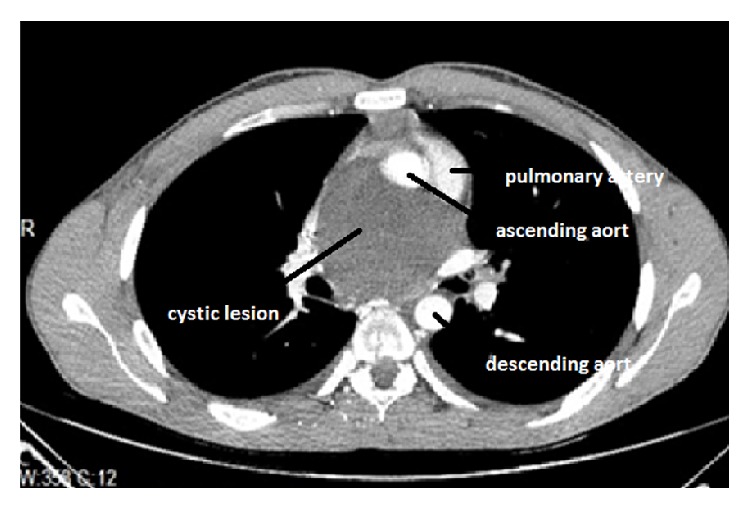
The computerized tomography of the patient showing 8 × 7 cm cystic structure situated above the right ventricle between the aortic arch and right pulmonary artery.

The patient was a 25-year-old male presented to the emergency department with dyspnea and swelling of his face and neck when he lay down. He complained of recent faint attacks in the last few weeks. Physical examination, chest radiography, and blood analysis were found to be normal. An echocardiography showed mild diastolic dysfunction and a cysts-like structure inside the pericardial cavity. The computerized tomography (CT) confirmed 8 × 7 cm cystic structure situated between the aortic arch and right pulmonary artery (Figure 1). We decided to surgically remove the mass lesion to treat myocardial diastolic dysfunction and for an accurate histopathologic diagnosis after the consent of the patient.

**Figure 2:**
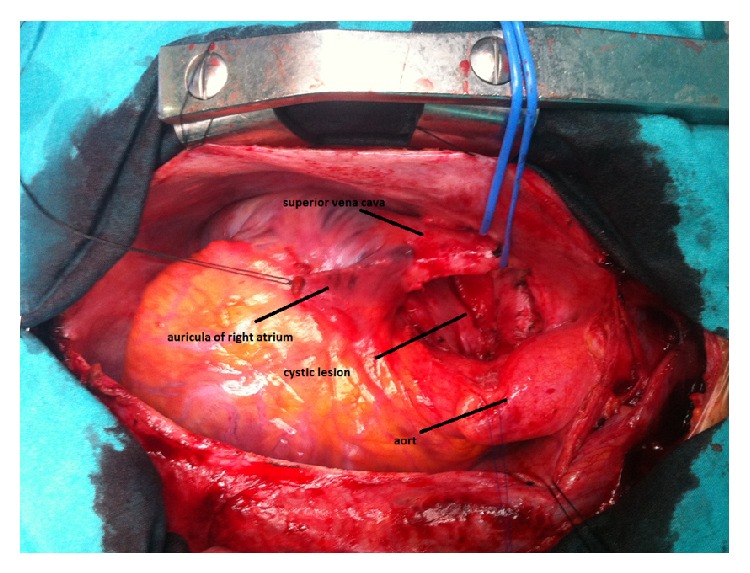
Perioperative view of the cyst.

**Figure 3:**
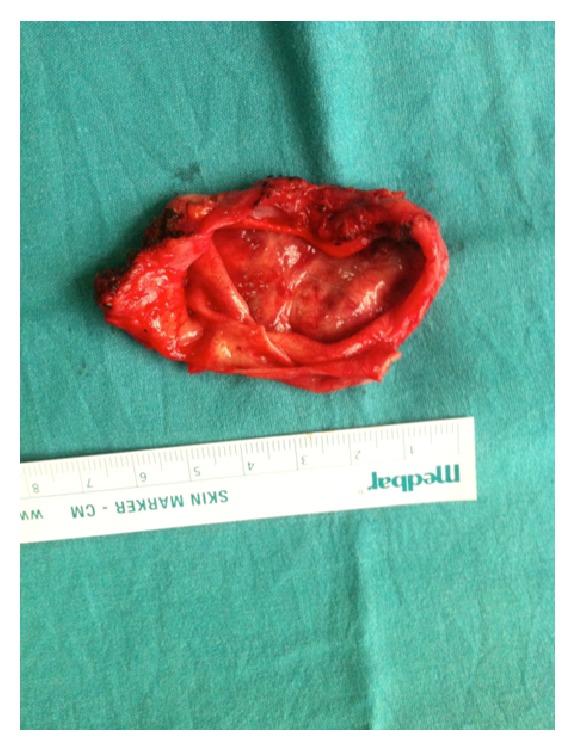
Resected cystic lesion.

Following standard median sternotomy, the pericardium was opened and 8 × 7 cm in diameter cystic structure was detected (Figure 2). After mobilizing the cyst from the right atrium, aorta, and the right pulmonary artery, it could be* en bloc* resected (Figure 3). The cyst was removed from the operating table to another table and cut. Yellow, jelly-like liquid was drained. Specimens for microbiology and histopatologic examination were obtained. A frozen section excluded malignancy and indicated a benign cystic lesion of bronchogenic origin. The cavity left after the removal of the cyst was covered with pericardium and the operation was finished uneventfully.

The patient was taken to the intensive care unit and weaned off mechanic ventilation in 6 hours. He was taken to the ward next day and discharged from the hospital on the postoperative 5th day. Microbiologic examination ended sterile and bronchogenic cyst diagnosis was confirmed with detailed histopathologic examination. The patient is followed regularly for more than 1 year at the outpatient clinic, and he was found to be asymptomatic throughout the followup period.

## 3. Discussion

Bronchogenic cysts are rare congenital lesions. They emerge due to abnormal development of the foregut which develops in a blind-end, fluid-filled pouch [4]. Although they are mostly located in the mediastinum, they may also occur in the lungs, diaphragm, retroperitoneum, pericardium, thymus, and neck [5]. Within the mediastinum, they are mainly situated at the subcarinal and right paratracheal regions [6]. Cases of intrapericardial and intracardiac bronchogenic cysts are very seldom in the literature [7]. Limaïem et al. reported an incidence of 1 case per 42,000 patients in the North American population [8].

Bronchogenic cysts usually do not cause symptoms and patients are mostly detected incidentally [5]. Very rarely, nonspecific symptoms such as retrosternal chest pain, dyspnea, cough, fever, and hoarseness may be present [6]. However, a huge cyst can provoke arterial compression, compression of the main bronchus and superior vena cava [5]. Although adults with cysts are usually symptomatic [5], in children they can cause symptoms due to the compression of the adjacent structures. Clinical findings such as cardiac murmur and electrocardiographic changes are described in a few cases in the literature [6].

Bronchogenic cysts are sacs filled with fluid. The radiologic diagnosis may be difficult because of air-fluid level or variable protein content as in case of an infection [5, 9]. As in our case, computerized tomography aids in preoperative diagnosis; however, differentiation between infections such as hydatid disease, a malignancy, or for the exact origin (i.e., pericardial, enteric, bronchogenic, or others) of the cyst can only be made with resection material and detailed histopathologic examination.

St-Georges et al. analyzed a collection of 86 resections of bronchogenic cysts. Their analysis revealed that 82% (the majority) of the patients became either symptomatic or complicated due to fistulization, ulceration, and infection in time [5]. McGlynn Jr. et al. reported that the cysts could even imitate an acute coronary syndrome [10]. Moreover, there are a few reports in the literature indicating malignancy potential of bronchogenic cysts, such as converting into large cell carcinoma [11], bronchoalveolar carcinoma [12], adenocarcinoma, and squamous cell carcinoma [13].

In conclusion, due to the proximity of the vital structures, surgical treatment is an approved, useful, and safe procedure with low complication rates for the treatment of bronchogenic cysts [14]. Considering the risk of complications and even malignancy, bronchogenic cysts require resection and should be performed when they are diagnosed.
